# Assessment of Time-Series Machine Learning Methods for Forecasting Hospital Discharge Volume

**DOI:** 10.1001/jamanetworkopen.2018.4087

**Published:** 2018-11-02

**Authors:** Thomas H. McCoy, Amelia M. Pellegrini, Roy H. Perlis

**Affiliations:** 1 Center for Quantitative Health, Department of Psychiatry, Massachusetts General Hospital, Harvard Medical School, Boston, Massachusetts

## Abstract

**Importance:**

Forecasting the volume of hospital discharges has important implications for resource allocation and represents an opportunity to improve patient safety at periods of elevated risk.

**Objective:**

To determine the performance of a new time-series machine learning method for forecasting hospital discharge volume compared with simpler methods.

**Design:**

A retrospective cohort study of daily hospital discharge volumes at 2 large, New England academic medical centers between January 1, 2005, and December 31, 2014 (hospital 1), or January 1, 2005, and December 31, 2010 (hospital 2), comparing time-series forecasting methods for prediction was performed. Data analysis was conducted from February 28, 2017, to August 30, 2018. Group-level data for all discharges from inpatient units were included. In addition to conventional methods, a technique originally developed for allocating data center resources, and comparison strategies for incorporating prior data and frequency of model updates, was conducted to identify the model application that optimized forecast accuracy.

**Main Outcomes and Measures:**

Model calibration as measured by *R*^2^ and, secondarily, number of days with errors greater than 1 SD of daily volume.

**Results:**

During the forecasted year, hospital 1 had 54 411 discharges (daily mean, 149) and hospital 2 had 47 456 discharges (daily mean, 130). The machine learning method was well calibrated at both sites (*R*^2^, 0.843 and 0.726, respectively) and made errors greater than 1 SD of daily volume on only 13 and 22 days, respectively, of the forecast year at the 2 sites. Last-value-carried-forward models performed somewhat less well (calibration *R*^2^, 0.781 and 0.596, respectively) with 13 and 46 errors of 1 SD or greater, respectively. More frequent retraining and training sets of longer than 1 year had minimal effects on the machine learning method’s performance.

**Conclusions and Relevance:**

Volume of hospital discharges can perhaps be reliably forecasted using simple carry-forward models as well as methods drawn from machine learning. The benefit of the latter does not appear to be dependent on extensive training data and may enable forecasts up to 1 year in advance with superior absolute accuracy to carry-forward models.

## Key Points

**Question:**

What is the performance of a new time-series machine learning method for predicting hospital discharge volume?

**Findings:**

In this cohort study of daily hospital discharge volumes at 2 academic medical centers (101 867 patient discharges), predictors of discharge volume were well calibrated. These findings were achieved even with shorter training sets and infrequent retraining.

**Meaning:**

These results appear to demonstrate the feasibility of deploying simple time-series methods to more precisely estimate hospital discharge volumes based on historical data, and may facilitate better matching of resources with clinical volume.

## Teaser

This cohort study assesses machine learning of time-series tools compared with simpler conventional methods to forecast the numbers of hospital discharges.

## Introduction

Variations in discharge volumes create a challenge for hospitals. Adequate staffing is essential for optimizing patient outcomes; however, these staff members are a significant source of fixed hospital cost.^1,2,3^ As such, volume-matched staffing is an important component in the goal of delivering high-value care. The biomedical literature includes many efforts to predict discharges at the level of hospital unit or clinical domain.^4,5,6^ Although these efforts are invaluable tools for discovery, the resource demand is such that they cannot typically be integrated into routine operations as a monitoring tool or scaled across all units; thus, there is a need for highly scalable forecasting approaches that are suitable for broad application and operational implementation.

Predicting time-series data—that is, using past information to forecast future values of the series—is an area of interest in the field of machine learning and statistics more broadly. Facebook recently released software implementing a Bayesian forecasting approach developed for allocation of computational resources.^7^ This method recognizes repeating patterns over weeks, months, years, and identified holidays. Recognizing that these secular trends are important drivers of hospital volume, we hypothesized that this method would also be well suited to hospital volume forecasting.

We further hypothesized that minimal dependence on tuning of hyperparameters, a challenge with many standard methods in machine learning, would make implementation practical and generalization possible. We therefore applied the Facebook forecasting method to predict discharge volume from 2 large academic medical centers. With an eye toward deployment of this system, we examined the importance of large training data sets (ie, considering longer vs shorter periods of time) and frequent training (ie, regenerating the model on a regular basis vs infrequently).^8,9^

The overall aim of the study was to understand this tool’s performance sufficiently to facilitate broader dissemination and application among hospital systems. To contextualize this understanding, we also applied simple previous-value-carried-forward and autoregressive approaches that have been studied by other investigators in the context of hospital volume forecasting.^10,11,12,13,14^

## Methods

### Overview and Data Set Generation

Hospital discharge data for each calendar date were extracted from the longitudinal electronic health records of 2 large, New England academic medical centers. Data covering different years were available from the 2 sites. At hospital 1, data from January 1, 2005, through December 31, 2014, were available, whereas at hospital 2, data from January 1, 2005, through December 31, 2010, were available. We analyzed time-series data in which the unit of analysis was calendar date. While hospital shifts do not correspond solely to such dates, the available data allowed reliable estimates of calendar dates only. No data were missing and, thus, no imputation strategy was required and all available data were included. Data analysis was conducted from February 28, 2017, to August 30, 2018. A datamart containing these data was generated with the i2b2, version 1.6 server software (i2b2 tranSMART Foundation), a computational framework for managing human health data.^15,16^

The Partners Human Research Committee approved all aspects of this study with waiver of informed consent. The study was conducted using the Strengthening the Reporting of Observational Studies in Epidemiology (STROBE) reporting guideline.

### Statistical Analysis

The primary learning task in this study was a forecast of daily hospital discharge volume for the last full year available for both hospitals (2010). This task was approached using 5 separate models for subsequent comparison: 3 simple variations on prior values carried forward, a seasonal autoregressive-integrated moving average (SARIMA) model, and Facebook’s Prophet model (FIS Corp).^7^ The primary outcome for comparison between models was prediction accuracy, measured by correlation between predicted value and actual observed value over the 1-year (2010) prediction horizon. This outcome was calculated as the linear model observed_day_ = β_0_ + β_1_forecasted_day,1_. As each component of this model is interpretable, it is reported in whole with *R*^2^ values and their 95% CIs.^17^ To further characterize model performance in units of discharges, error was operationalized as the difference of the predicted and the observed number of discharges over the forecast period (forecasted_day_ − observed_day_). Because the error can be negative and thus errors over the forecasting horizon could cancel one another, which may or may not be desirable depending on intended use, both total and total absolute error are reported.^18^ Except where noted in the secondary analysis, the forecasting horizon was 1 year.

Prophet is an open-source implementation (Python and R interfaces available) of a Bayesian forecaster with learned modeling of yearly and weekly seasonality, as well as prespecified holidays expected to be anomalous, which automatically detects change points in a growth curve, released by Facebook Research in early 2017. Conceptually, Prophet reframes forecasting as a curve fitting problem using a decomposable time-series model including holidays, seasonality, and overall trend that makes use of nonlinear smoothers.^19^

The 3 carry-forward models were the corresponding day, 1 year earlier; the corresponding day, 1 week earlier; and the mean of these 2. For example, for the yearly comparison, the second Monday of 2010 would be compared with the second Monday of 2009, representing a simple means of forecasting volume that still takes into account day of week and seasonal effects. For the weekly comparison, the second Monday of 2010 would be predicted to have the same volume as the first Monday of 2010. The third carry-forward forecast for the second week of 2010 would be the mean of the prior 2 (second Monday of 2009 and first Monday of 2010).

For the primary analysis, forecasting 2010 volume, Prophet was trained on all prior years (January 1, 2005, through December 31, 2009) and then used to predict the full 2010 calendar year. Hospital calendars were used to identify observed holidays at each site and these were used in training and forecasting of both the Prophet and SARIMA models. In all 5 cases, each hospital was modeled independently. All analysis was performed using R, version 3.4 with the R interface to Prophet, version 0.1.1.

### Model Parameter Investigation

We next examined 2 important operational characteristics of Prophet relevant to clinical dissemination and operationalization of hospital discharges forecasting. First, we allowed the training data set to vary between 1 and 5 years for all years at either site with at least 5 years of prior data available for training. In other words, as before, 2010 would be predicted but this time using first only 2009, then 2009 and 2008, then 2009 to 2007, and so on back to 2005. In this analysis, the years available for only 1 of the 2 hospitals (2011-2014) were included as forecasting targets, subject to the 5-year training data limit for comparability. This variable reflects the amount of training data required to build a reliable prediction model, that is, whether a hospital with a single year of discharge data could benefit from application of this model and whether a hospital could reasonably expect accuracy to improve with additional data. This assessment of the consequence of additional training data comes from the machine learning literature on learning curves.^20^

Second, we compared the forecast accuracy when run once a year vs rerunning on a monthly basis. In other words, as before, 2010 would be predicted but this time the first fit of the year (2005-2009) would be used to forecast January 2010; next, 2005 to January 2010 would be used to predict February 2010, and so on through the end of the year. This variable provides guidance about how frequently a model should be regenerated and insight into how quickly forecast accuracy degrades with distance from the last true observation. This iterative refitting of a model using a shorter forecasting horizon has conceptual validation to the idea of cross validation.^21^ These follow-up secondary experiments were performed only for the Prophet model.

## Results

**Figure 1.**
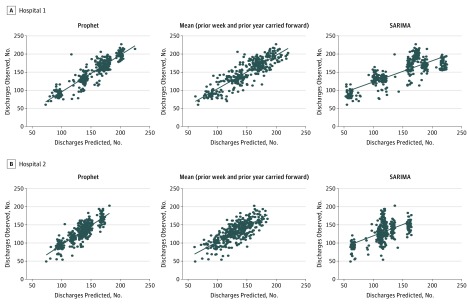
Comparison of Discharge Prediction Accuracy Through Calibration Curves for Prophet, Mean of Last Year and Last Week Carried Forward, and Seasonal Autoregressive Integrated Moving Average (SARIMA) Model

Over the course of the primary outcome year, 2010, hospital 1 had 54 411 discharges (daily mean, 149) and hospital 2 had 47 456 discharges (daily mean, 130). For the primary outcome, accuracy of the 2010 forecast based on all prior data, the Prophet model was the most accurate of the 5 models at both hospitals (Table 1 and Figure 1). The mean absolute error of the 1-year forecast by the Prophet model at hospital 1 was 11.5 discharges per day and 11.7 discharges per day at hospital 2. Among the 3 carry-forward models, the mean of the prior week and prior year’s value had the highest accuracy (Table 1). The mean absolute error of the forecast by the mean of the prior week and prior year carried forward model at hospital 1 was 13.7 discharges per day and 14.3 discharges per day at hospital 2. To further characterize the forecast accuracy, we selected 3 error thresholds (1 SD of daily volume, 25 discharges, and 10 discharges) and compared the total number of days for which the absolute forecast error was above the threshold for the 2 best models (Prophet and the mean of the prior week and year). These performance metrics are presented in Table 2, with Prophet outperforming the mean carry-forward model in 5 of 6 comparisons. Prophet was well calibrated at both sites (*R*^2^, 0.843 and 0.726, respectively) and made errors greater than 1 SD of daily volume on only 13 and 22 days, respectively, of the forecast year at the 2 sites. Last-value-carried-forward models performed somewhat less well (calibration *R*^2^, 0.781 and 0.596, respectively) with 13 and 46 errors of 1 SD or greater, respectively.

**Table 1.** Calibration of Target Year Prediction by Model and Hospital^a^

Model	Calibration (95% CI)	
	Hospital 1	Hospital 2
SARIMA	*y* = 64 + 0.58 × *x*; *R*^2^ = 0.655 (0.598-0.711)	*y* = 57 + 0.63 × *x*; *R*^2^ = 0.359 (0.281-0.437)
Last week carried forward	*y* = 30 + 0.8 × *x*; *R*^2^ = 0.644 (0.586-0.702)	*y* = 49 + 0.62 × *x*; *R*^2^ = 0.384 (0.306-0.461)
Last year carried forward	*y* = 21 + 0.86 × *x*; *R*^2^ = 0.756 (0.713-0.799)	*y* = 25 + 0.8 × *x*; *R*^2^ = 0.596 (0.532-0.659)
Mean of last week and year	*y* = 11 + 0.93 ×* x*; *R*^2^ = 0.781 (0.742-0.820)	*y* = 16 + 0.88 × *x*; *R*^2^ = 0.596 (0.532-0.659)
Prophet	*y* = −6.5 + 1 × *x*; *R*^2^ = 0.843 (0.814-0.872)	*y* = −13 + 1.1 × *x*; *R*^2^ = 0.726 (0.678-0.773)

Abbreviation: SARIMA, seasonal autoregressive integrated moving average.

^a^ With association calibrations shown in Figure 1.

**Table 2.** Number of Days Over Forecast Year With Forecast Error Exceeding a Given Threshold^a^

Error Threshold, Days	Days, No. (%)					
	Hospital 1			Hospital 2		
	Prophet Model	Mean of Last Week and Year	SARIMA	Prophet Model	Mean of Last Week and Year	SARIMA
>1 SD^b^	13 (3.56)	13 (3.56)	81 (22.19)	22 (6.03)	46 (12.6)	120 (32.89)
>25	28 (7.67)	56 (15.34)	173 (47.40)	32 (8.77)	59 (16.16)	142 (38.90)
>10	170 (46.58)	196 (53.7)	303 (83.01)	184 (50.41)	208 (56.99)	256 (70.14)

Abbreviation: SARIMA, seasonal autoregressive integrated moving average.

^a^ Denominator 365 days.

^b^ Standard deviation of each site’s daily discharge volume.

**Figure 2.**
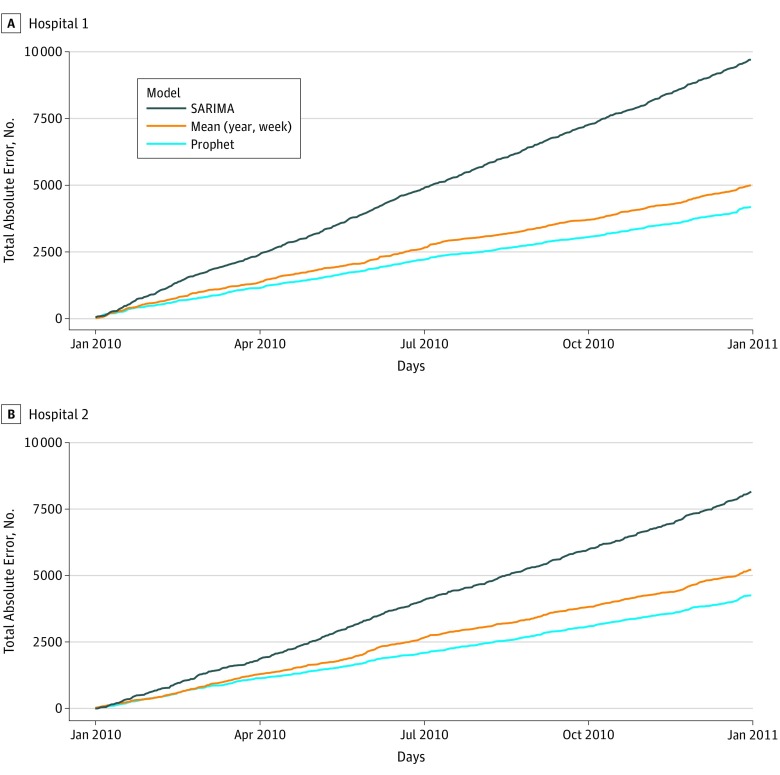
Comparison of Cumulative Total Absolute Error Over the Course of the Forecasted Year by Hospital Site and Predictive Model SARIMA indicates seasonal autoregressive integrated moving average.

We compared the total absolute forecast error and the total forecast error for both of the top-performing models (Table 3). In this comparison of total error, the mean carry-forward model outperformed Prophet on the net error over the course of the full year forecast, as this model tended to overpredict and underpredict in equal measure and thus negative and positive errors canceled each other over the course of the year, whereas Prophet consistently overpredicted hospital volume but did so to a lesser extent than the mean carry-forward model as indicated by the total absolute error in Table 3. Whether in terms of calibration (Table 1), days above error threshold (Table 2), or cumulative error over the full forecast horizon (Table 3 and Figure 2), the autoregressive model produced larger errors than the Prophet model.

**Table 3.** Absolute Total and Total Cumulative Error Over the Forecast Year^a^

Error Measure	Hospital 1 (n = 54 411)			Hospital 2 (n = 47 456)		
	Prophet Model	Mean of Last Week and Year	SARIMA	Prophet Model	Mean of Last Week and Year	SARIMA
Total absolute error	4189	4997	9699	4262	5220	8157
Total error	1295	−197	−1525	968	32	−5161

Abbreviation: SARIMA, seasonal autoregressive integrated moving average.

^a^ Denominator 365 days.

In the secondary analysis, we assessed the consequences of training data and forecast window on the accuracy of Prophet model predictions. Additional training data, added 1 year at a time, slightly increased the accuracy of Prophet forecasts and are summarized in eFigure 1 in the Supplement. Similarly, refitting the model monthly—using a shorter forecast horizon—had a minimal association with accuracy (eFigure 2 and eTables 1-3 in the Supplement, which mirror Table 1, Table 2, and Table 3 using the shorter prediction window).

## Discussion

In this effort to model volume of hospital discharge from 2 large academic medical centers spanning more than a decade, we found that an open-source tool intended to model server load reliably, if imprecisely, predicted volume. The predictions were better calibrated than those made by autoregressive models and simple carry forward of prior volumes. Moreover, the modest amount of training data required and the adequate performance for up to 365 days of follow-up suggest that this approach is feasible for essentially any hospital. It appears that the largest portion of forecast accuracy can be recognized with a single annual forecasting effort based on only the prior year’s data. Unlike many methods in machine learning, the model training and forecasting reported herein can be replicated on an Intel i5-2400 system from 2011 in less than half an hour. In short, this method is neither data nor compute intensive and thus could be widely adopted. As such, given that the existing literature on forecasting using carry-forward models, conventional regression, autoregression, and more exotic models is mixed with respect to most successful model, the Prophet model is of particular appeal as it both performs well and is highly usable in terms of computational, data, and human resources.^10,11,12,13,14,22^

Is the ability to reliably predict volume useful for quality and safety? Certainly at the extremes, matching patient load is important; studies suggest optimal patient to clinical staff ratios vary substantially by specialty but are associated with a range of outcomes, including mortality.^23^ Differences in risk and length of stay associated with discharge on weekends or at night further underscore the importance of such staffing decisions, although not all studies find such variability.^24,25,26,27,28^ Conversely, consistently erring on the side of overstaffing is likely to entail additional costs, consuming resources that could be better spent on other quality-improvement strategies. As such, even coarse predictions may allow hospital administrators to better balance staffing and patient needs. We are not the first to note the importance of holidays in forecasting hospital volume as these days are of particular relevance in staffing.^13^ Furthermore, we are interested in the possibility of using real-time deviation from forecasted volume at the nursing unit and clinical service level as a means of gaining insight into health system performance; however, this application requires additional work beyond the foundational effort reported here.

### Limitations

We note several limitations in interpreting these results. First, while on average, errors are small, the absolute errors on any given day may be relatively large. At each of the 2 hospitals, the error exceeded 25 patients on fewer than 10% of the days. Although these errors are still less than those arising from a simpler prediction approach, they nonetheless indicate that a flexible staffing model is likely to be necessary even with optimal prediction. In addition, we emphasize that these estimates represent only a starting point. It is likely that further optimization, for example, taking into account weather or local rates of influenza infection in winter, or modeling individual units, would allow more precise near-term predictions.^12^ On the other hand, a strength of the approach studied here is that it is readily implemented at nearly any site without requiring other data streams or tuning of hyperparameters. The ease of fitting is of particular importance given the variability in model performance seen between the 2 hospital sites. This variability is consistent with the existing literature that shows variable results.^10,11,12,13,14^ As such, those looking to forecast volume should evaluate a range of models and consider adding additional variables beyond historical volume if forecasts are of insufficient accuracy.

We note an important principle of forecasting in general: these tools are best applied thoughtfully, with consideration of their strengths and limitations. For example, computers cannot be expected to incorporate externalities unavailable to them, such as changes in patient flow related to the availability of beds at other hospitals or to reimbursement.

## Conclusions

For all the enthusiasm about machine learning in medicine, which seems to recur approximately every 30 years,^29^ impact on real-world clinical practice remain modest; a recent commentary noted the mismatch between promise and concrete accomplishment.^30^ The present study suggests that straightforward application of existing software would allow reliable prediction of a critically important metric of hospital operation and that such application need not use prohibitively large data sets, computational resources, or the operational complexity of frequent updates. While more advanced models are developed, time-series–based prediction offers the possibility of improving clinical planning in the near term.

## Notes

Supplement. **eFigure 1.** Learning Curve for Each of Six Target Years Showing Impact of Additional Years of Training Data on Mean Forecast Accuracy and Min and Max Forecast Accuracy **eFigure 2.** Prediction Calibration by Hospital Site for the Prophet Model Using a Full One-Year Forecasting Horizon vs a Monthly Forecasting Horizon **eTable 1.** Model Calibrations for Full Year Forecast Horizon vs Iterative Refitting With a Monthly Forecast Horizon (Full Year Is Reproduced From Table 1 for Ease of Comparison) **eTable 2.** Number of Days Over Forecast Year by Error Sizes for Prophet Model Using Full Year Forecast Horizon (Reproduced From Table 2) vs Monthly Stepwise Forecast Horizon **eTable 3.** Absolute Total and Total Cumulative Error Over the Forecast Year (Denominator 365 Days) Comparing Full Year Forecast Horizon and Iterative Monthly Forecast Horizon

## References

[1] Beltempo M, Blais R, Lacroix G, Cabot M, Piedboeuf B Association of nursing overtime, nurse staffing, and unit occupancy with health care–associated infections in the NICU. Am J Perinatol. 2017;34(10):-. doi:10.1055/s-0037-1601459 28376546

[2] Griffiths P, Ball J, Drennan J, Nurse staffing and patient outcomes: strengths and limitations of the evidence to inform policy and practice—a review and discussion paper based on evidence reviewed for the National Institute for Health and Care Excellence Safe Staffing guideline development. Int J Nurs Stud. 2016;63:213-225. doi:10.1016/j.ijnurstu.2016.03.012 27130150

[3] Kim Y, Kim S-H, Ko Y Effect of nurse staffing variation and hospital resource utilization. Nurs Health Sci. 2016;18(4):473-480. doi:10.1111/nhs.12294 27396974

[4] DeRienzo CM, Shaw RJ, Meanor P, Lada E, Ferranti J, Tanaka D A discrete event simulation tool to support and predict hospital and clinic staffing. Health Informatics J. 2017;23(2):124-133. doi:10.1177/1460458216628314 26928193

[5] Schreyer KE, Martin R The economics of an admissions holding unit. West J Emerg Med. 2017;18(4):553-558. doi:10.5811/westjem.2017.4.32740 28611873 PMC5468058

[6] Gardner G, Gardner A, Middleton S, Mapping workforce configuration and operational models in Australian emergency departments: a national survey. Aust Health Rev. 2018;42(3):340-347. doi:10.1071/AH16231 28514641

[7] Taylor SJ, Letham B Prophet: forecasting at scale. Facebook Research. https://research.fb.com/prophet-forecasting-at-scale. Published February 23, 2017. Accessed August 30, 2018.

[8] Friedman JH On bias, variance, 0/1—loss, and the curse-of-dimensionality. Data Min Knowl Discov. 1997;1(1):55-77. doi:10.1023/A:1009778005914

[9] Geman S, Bienenstock E, Doursat R Neural networks and the bias/variance dilemma. Neural Comput. 1992;4(1):1-58. doi:10.1162/neco.1992.4.1.1

[10] Tandberg D, Qualls C Time series forecasts of emergency department patient volume, length of stay, and acuity. Ann Emerg Med. 1994;23(2):299-306. doi:10.1016/S0196-0644(94)70044-3 8304612

[11] Bergs J, Heerinckx P, Verelst S Knowing what to expect, forecasting monthly emergency department visits: A time-series analysis. Int Emerg Nurs. 2014;22(2):112-115. doi:10.1016/j.ienj.2013.08.001 24055373

[12] Jones SS, Thomas A, Evans RS, Welch SJ, Haug PJ, Snow GL Forecasting daily patient volumes in the emergency department. Acad Emerg Med. 2008;15(2):159-170. doi:10.1111/j.1553-2712.2007.00032.x 18275446

[13] Luo L, Xu X, Li J, Shen W Short-term forecasting of hospital discharge volume based on time series analysis. In: 2017 IEEE 19th International Conference on E-Health Networking, Applications and Services (Healthcom). Dalian, China: IEEE; 2017:1-6. doi:10.1109/HealthCom.2017.8210801

[14] Ting Zhu, Li Luo, Xinli Zhang, Yingkang Shi, Wenwu Shen Time-series approaches for forecasting the number of hospital daily discharged inpatients. IEEE J Biomed Health Inform. 2017;21(2):515-526. doi:10.1109/JBHI.2015.2511820 28055928

[15] Murphy SN, Mendis M, Hackett K, Architecture of the open-source clinical research chart from Informatics for Integrating Biology and the Bedside. AMIA Annu Symp Proc. 2007:548-552. 18693896 PMC2655844

[16] Murphy SN, Weber G, Mendis M, Serving the enterprise and beyond with informatics for integrating biology and the bedside (i2b2). J Am Med Inform Assoc. 2010;17(2):124-130. doi:10.1136/jamia.2009.000893 20190053 PMC3000779

[17] Cohen J, Cohen P, West SG, Aiken LS Applied Multiple Regression/Correlation Analysis for the Behavioral Sciences. 3rd ed Mahwah, NH: Lawrence Erlbaum Associates Inc; 2002.

[18] De Gooijer JG, Hyndman RJ 25 years of time series forecasting. Int J Forecast. 2006;22(3):443-473. doi:10.1016/j.ijforecast.2006.01.001

[19] Taylor SJ, Letham B Forecasting at scale. PeerJ Preprints. https://peerj.com/preprints/3190/. Published September 27, 2017. Accessed August 30, 2018. doi:10.7287/peerj.preprints.3190v2

[20] Perlich C, Provost F, Simonoff JS Tree induction vs logistic regression: a learning-curve analysis. J Mach Learn Res. 2003;4:211-255. doi:10.1162/153244304322972694

[21] Bergmeir C, Hyndman RJ, Koo B A note on the validity of cross-validation for evaluating autoregressive time series prediction. Comput Stat Data Anal. 2018;120:70-83. doi:10.1016/j.csda.2017.11.003

[22] Kim K, Lee C, O’Leary KJ, Rosenauer S, Mehrotra S Predicting Patient Volumes in Hospital Medicine: A Comparative Study of Different Time Series Forecasting Methods. Evanston, IL: Northwestern University; 2014:1-13.

[23] Gershengorn HB, Harrison DA, Garland A, Wilcox ME, Rowan KM, Wunsch H Association of intensive care unit patient-to-intensivist ratios with hospital mortality. JAMA Intern Med. 2017;177(3):388-396. doi:10.1001/jamainternmed.2016.8457 28118657

[24] Keswani A, Beck C, Meier KM, Fields A, Bronson MJ, Moucha CS Day of surgery and surgical start time affect hospital length of stay after total hip arthroplasty. J Arthroplasty. 2016;31(11):2426-2431. doi:10.1016/j.arth.2016.04.013 27491449

[25] de Cordova PB, Johansen ML, Martinez ME, Cimiotti JP Emergency department weekend presentation and mortality in patients with acute myocardial infarction. Nurs Res. 2017;66(1):20-27. doi:10.1097/NNR.0000000000000196 27977565

[26] Hinds N, Borah A, Yoo EJ Outcomes of nighttime refusal of admission to the intensive care unit: the role of the intensivist in triage. J Crit Care. 2017;39:214-219. doi:10.1016/j.jcrc.2016.12.024 28279496

[27] Metcalfe D, Perry DC, Bouamra O, Is there a “weekend effect” in major trauma? Emerg Med J. 2016;33(12):836-842. doi:10.1136/emermed-2016-206049 27789565

[28] Hamaguchi S, Kinugawa S, Tsuchihashi-Makaya M, Goto D, Tsutsui H Weekend versus weekday hospital admission and outcomes during hospitalization for patients due to worsening heart failure: a report from Japanese Cardiac Registry of Heart Failure in Cardiology (JCARE-CARD). Heart Vessels. 2014;29(3):328-335. doi:10.1007/s00380-013-0359-5 23653107

[29] Schwartz WB, Patil RS, Szolovits P Artificial intelligence in medicine: where do we stand? N Engl J Med. 1987;316(11):685-688. doi:10.1056/NEJM198703123161109 3821801

[30] Chen JH, Asch SM Machine learning and prediction in medicine—beyond the peak of inflated expectations. N Engl J Med. 2017;376(26):2507-2509. doi:10.1056/NEJMp1702071 28657867 PMC5953825

