# Supplementary material for: Assessment of Time-Series Machine Learning Methods for Forecasting Hospital Discharge Volume
Source: JAMA Netw Open. 2018 Nov 2;1(7):e184087. doi: 10.1001/jamanetworkopen.2018.4087 (PMC6324591; doi:10.1001/jamanetworkopen.2018.4087)
Supplement: Supplement. — eFigure 1. Learning Curve for Each of Six Target Years Showing Impact of Additional Years of Training Data on Mean Forecast Accuracy and Min and Max Forecast Accuracy eFigure 2. Prediction Calibration by Hospital Site for the Prophet Model Using a Full One-Year Forecasting Horizon vs a Monthly Forecasting Horizon eTable 1. Model Calibrations for Full Year Forecast Horizon vs Iterative Refitting With a Monthly Forecast Horizon (Full Year Is Reproduced From Table 1 for Ease of Comparison) eTable 2. Number of Days Over Forecast Year by Error Sizes for Prophet Model Using Full Year Forecast Horizon (Reproduced From Table 2) vs Monthly Stepwise Forecast Horizon eTable 3. Absolute Total and Total Cumulative Error Over the Forecast Year (Denominator 365 Days) Comparing Full Year Forecast Horizon and Iterative Monthly Forecast Horizon [file jamanetwopen-1-e184087-s001.pdf]

## Supplementary Online Content

McCoy TH Jr, Pellegrini AM, Perlis RH. Assessment of time-series machine learning methods for forecasting hospital discharge volume. *JAMA Netw Open*. 2018;1(7):e184087. doi:10.1001/jamanetworkopen.2018.4087

**eFigure 1.** Learning Curve for Each of Six Target Years Showing Impact of Additional Years of Training Data on Mean Forecast Accuracy and Min and Max Forecast Accuracy

**eFigure 2.** Prediction Calibration by Hospital Site for the Prophet Model Using a Full One-Year Forecasting Horizon vs a Monthly Forecasting Horizon

**eTable 1.** Model Calibrations for Full Year Forecast Horizon vs Iterative Refitting With a Monthly Forecast Horizon (Full Year Is Reproduced From Table 1 for Ease of Comparison)

**eTable 2.** Number of Days Over Forecast Year by Error Sizes for Prophet Model Using Full Year Forecast Horizon (Reproduced From Table 2) vs Monthly Stepwise Forecast Horizon

**eTable 3.** Absolute Total and Total Cumulative Error Over the Forecast Year (Denominator 365 Days) Comparing Full Year Forecast Horizon and Iterative Monthly Forecast Horizon

This supplementary material has been provided by the authors to give readers additional information about their work.

© 2018 McCoy TH Jr et al. *JAMA Network Open.*

**eFigure 1.** Learning Curve for Each of Six Target Years Showing Impact of Additional Years of Training Data on Mean Forecast Accuracy (dot) and Min And Max Forecast Accuracy (whisker)

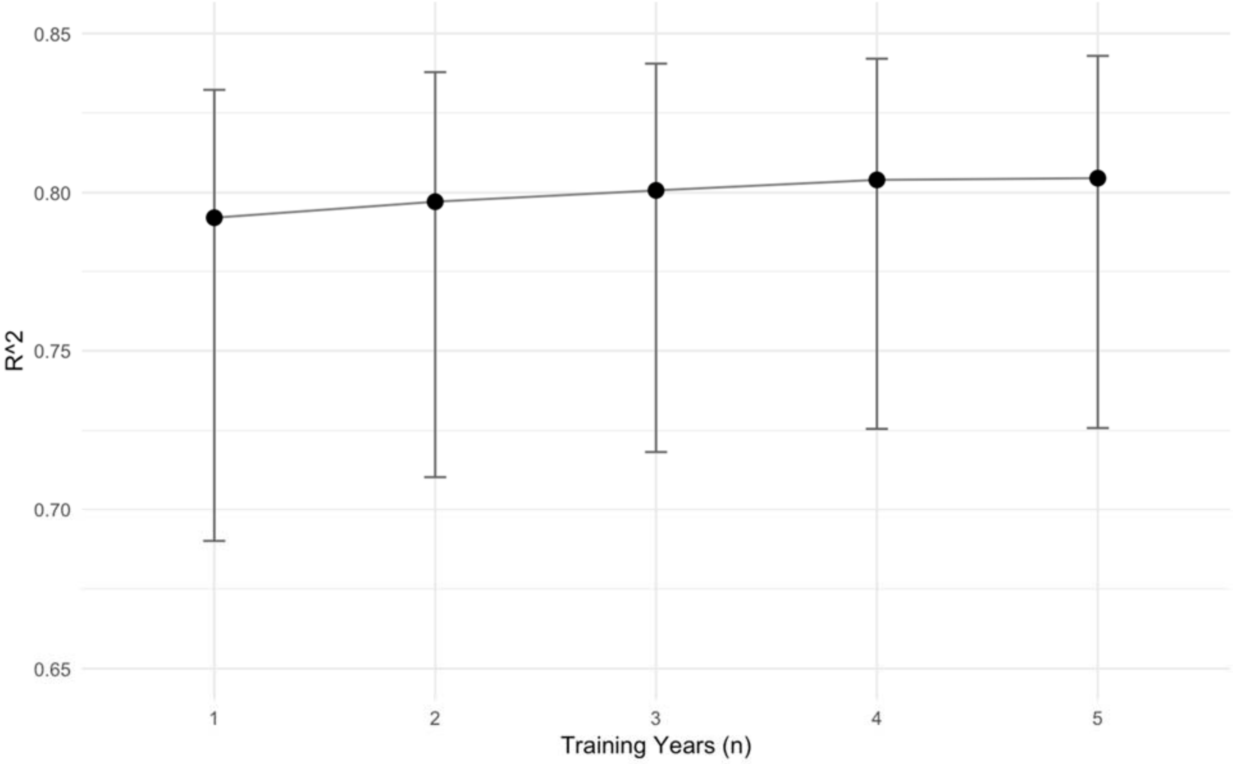


© 2018 McCoy TH Jr et al. *JAMA Network Open.*

**eFigure 2.** Prediction Calibration by Hospital Site for the Prophet Model Using a Full One-Year Forecasting Horizon vs a Monthly Forecasting Horizon

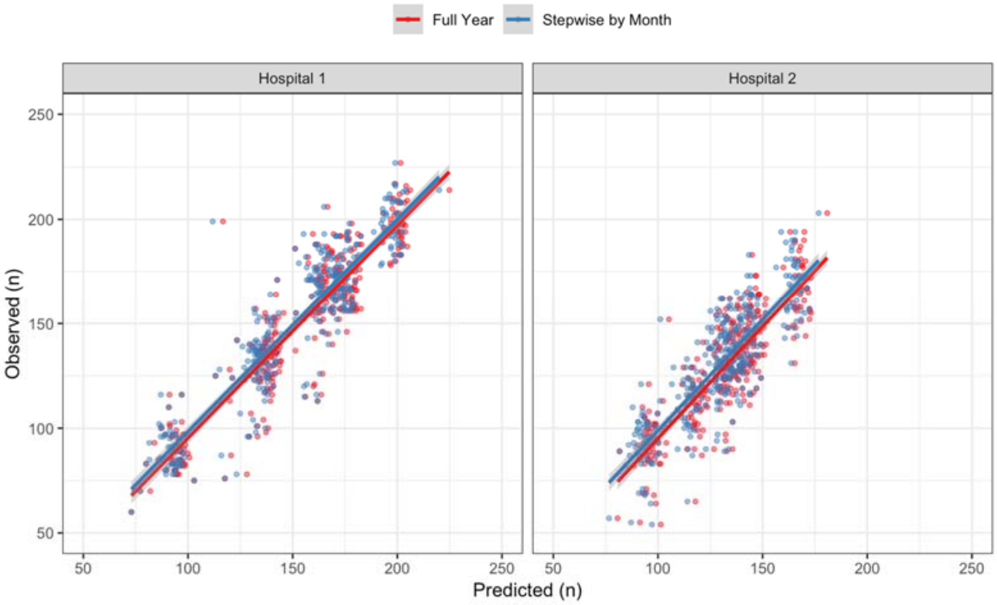


© 2018 McCoy TH Jr et al. *JAMA Network Open.*

**eTable 1.** Model Calibrations for Full Year Forecast Horizon vs Iterative Refitting With a Monthly Forecast Horizon (Full Year Is Reproduced From Table 1 for Ease of Comparison)

| **Prophet Forecast Horizon** | **Hospital 1** | **Hospital 2** |
|---|---|---|
| full year | $y = -6.5 + 1 \times x$; $r^2 = 0.843$ | $y = -13 + 1.1 \times x$; $r^2 = 0.726$ |
| monthly | $y = -3.2 + 1 \times x$; $r^2 = 0.841$ | $y = -8.6 + 1.1 \times x$; $r^2 = 0.717$ |

© 2018 McCoy TH Jr et al. *JAMA Network Open.*

**eTable 2.** Number of Days Over Forecast Year by Error Sizes for Prophet Model Using Full Year Forecast Horizon (Reproduced From Table 2) vs Monthly Stepwise Forecast Horizon

| | **Hospital 1** | | **Hospital 2** | |
|---|---|---|---|---|
| **Error Threshold** | Full Year | By Month | Full Year | By Month |
| >1 SD | 13 | 8 | 22 | 25 |
| >25 | 28 | 31 | 32 | 36 |
| >10 | 170 | 159 | 184 | 170 |

© 2018 McCoy TH Jr et al. *JAMA Network Open.*

**eTable 3.** Absolute Total and Total Cumulative Error Over the Forecast Year (Denominator 365 Days) Comparing Full Year Forecast Horizon and Iterative Monthly Forecast Horizon

| | **Hospital 1** (n= 54411) | | **Hospital 2** (n= 47456) | |
|---|---|---|---|---|
| **Error measure** | Full Year | By Month | Full Year | By Month |
| Total Absolute Error | 4189 | 4015 | 4262 | 4201 |
| Total Error | 1295 | 292 | 968 | -204 |

© 2018 McCoy TH Jr et al. *JAMA Network Open.*
